# Ist2 in the Yeast Cortical Endoplasmic Reticulum Promotes Trafficking of the Amino Acid Transporter Bap2 to the Plasma Membrane

**DOI:** 10.1371/journal.pone.0085418

**Published:** 2014-01-08

**Authors:** Wendelin Wolf, Klaus Meese, Matthias Seedorf

**Affiliations:** Zentrum für Molekulare Biologie der Universität Heidelberg (ZMBH), DKFZ-ZMBH-Allianz, Heidelberg, Germany; Texas A&M University, United States of America

## Abstract

The equipment of the plasma membrane in *Saccharomyces cerevisiae* with specific nutrient transporters is highly regulated by transcription, translation and protein trafficking allowing growth in changing environments. The activity of these transporters depends on a H^+^ gradient across the plasma membrane generated by the H^+^-ATPase Pma1. We found that the polytopic membrane protein Ist2 in the cortical endoplasmic reticulum (ER) is required for efficient leucine uptake during the transition from fermentation to respiration. Experiments employing tandem fluorescence timer protein tag showed that Ist2 was necessary for efficient trafficking of newly synthesized leucine transporter Bap2 from the ER to the plasma membrane. This finding explains the growth defect of *ist2*Δ mutants during nutritional challenges and illustrates the important role of physical coupling between cortical ER and plasma membrane.

## Introduction

Eukaryotic cells regulate growth and proliferation in response to environmental changes. This involves the regulation of the activity and abundance of receptors and transporters in their plasma membrane (PM). Amino acid transporters use the H^+^-gradient across the PM as a driving force for amino acid uptake. This gradient is generated by the abundant H^+^-ATPase Pma1, which pumps H^+^ across the PM, acidifies the growth medium and contributes to the control of cytosolic pH [1]. Abundance control of amino acid transporters in *Saccharomyces cerevisiae* involves regulation at all levels from transcription to protein synthesis, protein trafficking and protein stability. Several amino acid transporters are induced by the presence of their substrate amino acids and delivered to the PM when amino acids are present in the growth media [2], while absence of substrates and/or stress causes down-regulation via endocytosis and delivery to the vacuole [3], [4]. Another aspect of this regulation is the specialization of the PM into distinct and stable domains [5], [6]. One of these domains is the membrane compartment of Can1 (MCC), where the arginine transporter Can1 accumulates in dot-like structures [7].

MCC localization is essential for Can1 function [6], suggesting that localization in PM subdomains and therefore interaction with associated membranes determines protein function. Between 20 and 45% of the yeast PM is tightly associated with cortical ER, with an average distance of 33 nm between both membranes [8]. Whether the cortical ER influences domain organisation and function of PM proteins, their endocytosis rates or signalling processes remain open.

ER-PM tethering depends on a number of proteins in the cortical ER: the VAP orthologs Scs2 and Scs22, the extended synaptotagmin orthologs Tcb1 and Tcb3, and the putative member of the ANO/TMEM16 ion channel family Ist2 [9]. The tail-anchored proteins Scs2 and Scs22 bind lipid transfer proteins and have been implicated in Sac1-mediated phosphoinositide (PI) 4-phosphate (PI(4)P) turnover [10], [11]. The tricalbins Tcb1 and Tcb3 are integral membrane proteins with a cytosolically exposed PI(4,5)P_2_ binding domain of the C2 type [12] and mammalian orthologs contribute to ER-PM contact formation [13]. Ist2 is the only member of the ANO/TMEM16 protein family in *Saccharomyces cerevisiae*, of which some mammalian orthologs function as Ca^2+^-regulated ion channels of the PM [14]–[17]. Similar to the tricalbins, Ist2 employs a cytosolically exposed lysine-rich domain for binding PI(4,5)P_2_ at the PM and ER-PM tethering [18]–[20]. Deletion of *IST2* results in increased distance between cortical ER and PM and as a consequence ribosomes gain access to the narrow space between cortical ER and PM, while they are excluded from this area in WT [8], [20].

Our previous study revealed that deletion of *IST2* affects the growth rate after dilution of cells into fresh synthetic media [20]. Moreover, we found that Ist2 contributes to glucose-stimulated H^+^ pumping across the PM, suggesting a role of Ist2 as an ion channel of the cortical ER supporting the function of Pma1. Alternatively, Ist2-mediated ER-PM tethering could influence exo- and endocytosis rates and/or cellular signaling that depends on ER-PM tethering. By careful analysis of the *ist2*Δ growth phenotype, we found a requirement of Ist2 for efficient trafficking of newly synthesized leucine transporter Bap2 from the ER to the PM.

## Results

### Ist2 is Required for Fast Growth Under Changing Nutrient Supply

**Figure 1:**
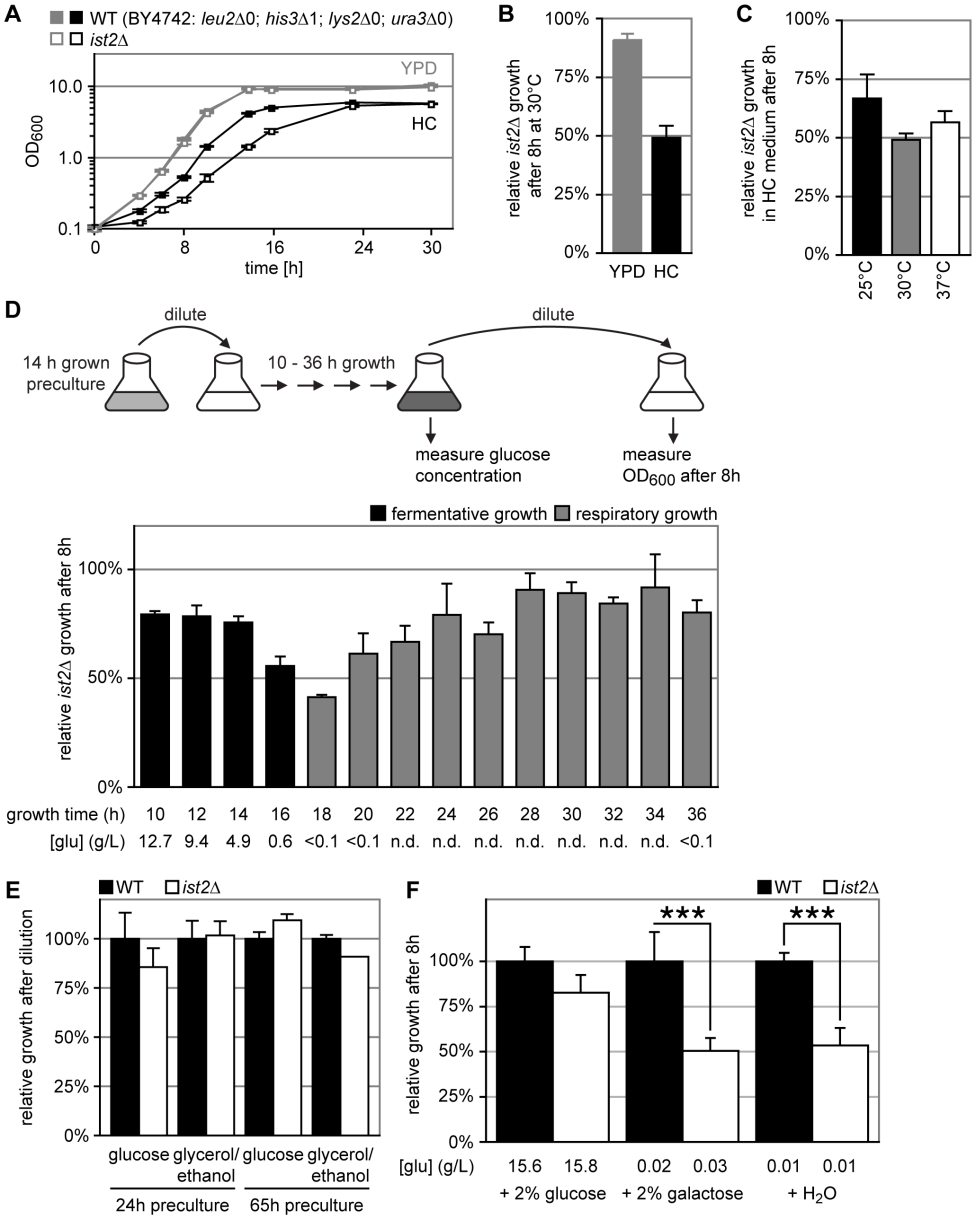
Transition from fermentation to respiration is impaired in *ist2*Δ. (A) WT (BY4742 = *leu2*Δ*0, his3*Δ*1, lys2*Δ*0, ura3*Δ*0*; closed squares) and *ist2*Δ (open squares) cells were grown in YPD (grey) or HC (black) media at 30°C. Cells were diluted to 0.1 OD_600_ from pre-cultures grown in HC for 24 hours at 25°C. (B) Growth of *ist2*Δ cells relative to WT ( = 100%) 8 h after dilution into YPD (grey) and HC (black) media. (C) Growth of *ist2*Δ cells relative to WT ( = 100%) at 25°C (black), 30°C (grey) or 37°C (white) 8 h after dilution into HC medium. (D) WT and *ist2*Δ cells were pre-cultured for 14 hours in HC medium to 2.60±0.94 and 3.50±0.94 OD_600_, respectively and inoculated to 0.1 OD_600_ into fresh HC medium. At the indicated time points the glucose concentration (g/L) of the medium was determined. Additionally, cells were diluted to 0.1 OD_600_ into fresh HC medium, grown for 8 hours and relative growth of *ist2*Δ cells was determined (see cartoon for overview; pc: pre-culture). Growth phase was classified as respiratory (grey) when media glucose concentration was <0.1 g/L. Error bars indicate s.d.m., n = 3. (E) WT (black) and *ist2*Δ (white) cells were grown in HC media containing 3% glycerol and 2% ethanol as sole carbon source for 24 hours to 2.4±0.7 and 2.4±0.7 OD_600_ or for 65 hours to 10.9±0.7 and 11.2±0.3 OD_600_, respectively. Cells were diluted to 0.1 OD_600_ into fresh HC media containing 2% glucose or 3% glycerol and 2% ethanol and grown for 8 hours. OD_600_ was normalized to the respective WT and is shown as mean+s.d.m. (n = 6 for 24 hours pre-cultures and n = 3 for 65 hours pre-cultures). (F) WT and *ist2*Δ cells were grown in HC medium with 2% glucose for 18 hours until glucose depletion. Cultures were supplemented with 2% glucose, 2% galactose or mock treated with water for 30 min and diluted to 0.1 OD_600_ into HC medium containing 2% glucose and grown for 8 hours. OD_600_ was normalized to the respective WT. Error bars indicate s.d.m (n ≥5). The glucose concentration was measured after 30 min of treatment. Triple asterisks indicate significance of p<0.001 (unpaired, two-tailed student’s t-test).

In order to gain insight into the physiological function of Ist2, we compared the growth of BY4742 wild type (WT) and *ist2*Δ strains. Dilution of pre-cultures that have reached stationary phase into YPD media resulted in similar growth of isogenic WT and *ist2*Δ strains, while dilution into synthetic Hartwell’s Complete (HC) media led to delayed growth of *ist2*Δ cells (Fig. 1A). To better resolve this growth delay, we measured OD_600_ 8 hours after dilution. Compared to WT the density of *ist2*Δ cells was 49±2.7% in HC media and 90.7% ±5.2% in YPD media at 30°C (Fig. 1B). This *ist2*Δ–specific growth delay occurred at 25, 30, and 37°C but was most pronounced at 30°C (Fig. 1C). To ask whether reduced viability caused this growth delay, we analyzed colony formation of individual cells. More than 80% of *ist2*Δ and WT cells formed large colonies, while 11% of *ist2*Δ and 5% of WT cells formed small colonies (Fig. S1A). Thus reduced viability cannot explain the observed growth delay. To analyze whether *ist2*Δ cells display a prolonged lag phase, we measured OD_600_ between 3 and 6 hours after dilution. Both *ist2*Δ and WT cells resumed growth 3 to 3.5 hours after dilution, followed by reduced initial growth rate of *ist2*Δ (Fig. S1B). Taken together, this indicates that growth of *ist2*Δ is homogenously delayed. Decreased viability and prolonged lag phase are not a major cause of the *ist2*Δ phenotype.

Our previous work revealed that the growth delay of *ist2*Δ cells was restricted to the dilution of cells that are entering stationary phase and we observed no effect upon dilution of log-phase cells [20], suggesting that the consumption of glucose and/or other nutrients in synthetic HC media caused this phenotype. First, we asked whether the consumption of glucose correlates with the onset of the *ist2*Δ growth delay. Such a change in metabolism from mainly aerobic fermentation in presence of glucose to respiration is known as diauxic shift and involves large and rapid adaptation in cellular gene expression [21], [22]. The *ist2*Δ growth delay was most pronounced upon dilution of cells grown for 18 hours (Fig. 1D). At this time point glucose concentration in the media dropped below 0.1 g/L, indicating that these cells have consumed most glucose and entered the diauxic shift. This transition correlated with slower growth of *ist2*Δ and WT as compared to cells diluted from log phase (Fig. S1C). Dilution of pre-cultures grown for 24 hours or longer led to accelerated growth (Fig. S1C) and *ist2*Δ cells reached more than 70% of WT cell density (Fig. 1D and E), confirming that the growth delay of *ist2*Δ is most prominent during the transition from fermentation to respiration. Consistently, the dilution of respiring *ist2*Δ and WT cells (pre-cultures grown for 24 or 65 hours in 2% ethanol and 3% glycerol) into fresh HC media containing either glucose or ethanol/glycerol led to similar growth of both *ist2*Δ and WT (Fig. 1E). In order to test whether the consumption of glucose caused the *ist2*Δ growth delay, we grew pre-cultures for 18 hours and added 2% glucose or galactose for 30 min prior to dilution. This maintains low nutrient concentrations in the media but replenishes glucose to 20 g/L. Glucose addition improved growth of *ist2*Δ and these cultures reached 82.7±9.8% of WT density, while galactose or H_2_O addition had no effect on the *ist2*Δ phenotype (Fig. 1F). Taken together, Ist2 is required for adaptation to glucose depletion and the transition from fermentation to respiration. Since 30 min glucose addition prior to dilution was sufficient to rescue the growth delay of *ist2*Δ, these data suggest that the defect in *ist2*Δ is compensated by rapid activation of glucose signalling [23].

### Acidification of the Cytosol Correlates with the Ist2-dependent Growth Phenotype

Glucose depletion reduces Pma1 activity [24] and Ist2 as a putative ion channel [20] may support the formation of H^+^ gradients locally. Therefore, low Pma1 activity and a compromised H^+^-gradient as consequence of glucose depletion may explain why the *ist2*Δ phenotype was most prominent during the transition from fermentation to respiration. In order to test this idea, we followed how the transition from fermentation to respiration affected the intracellular pH in both cytosol and ER lumen.

**Figure 2:**
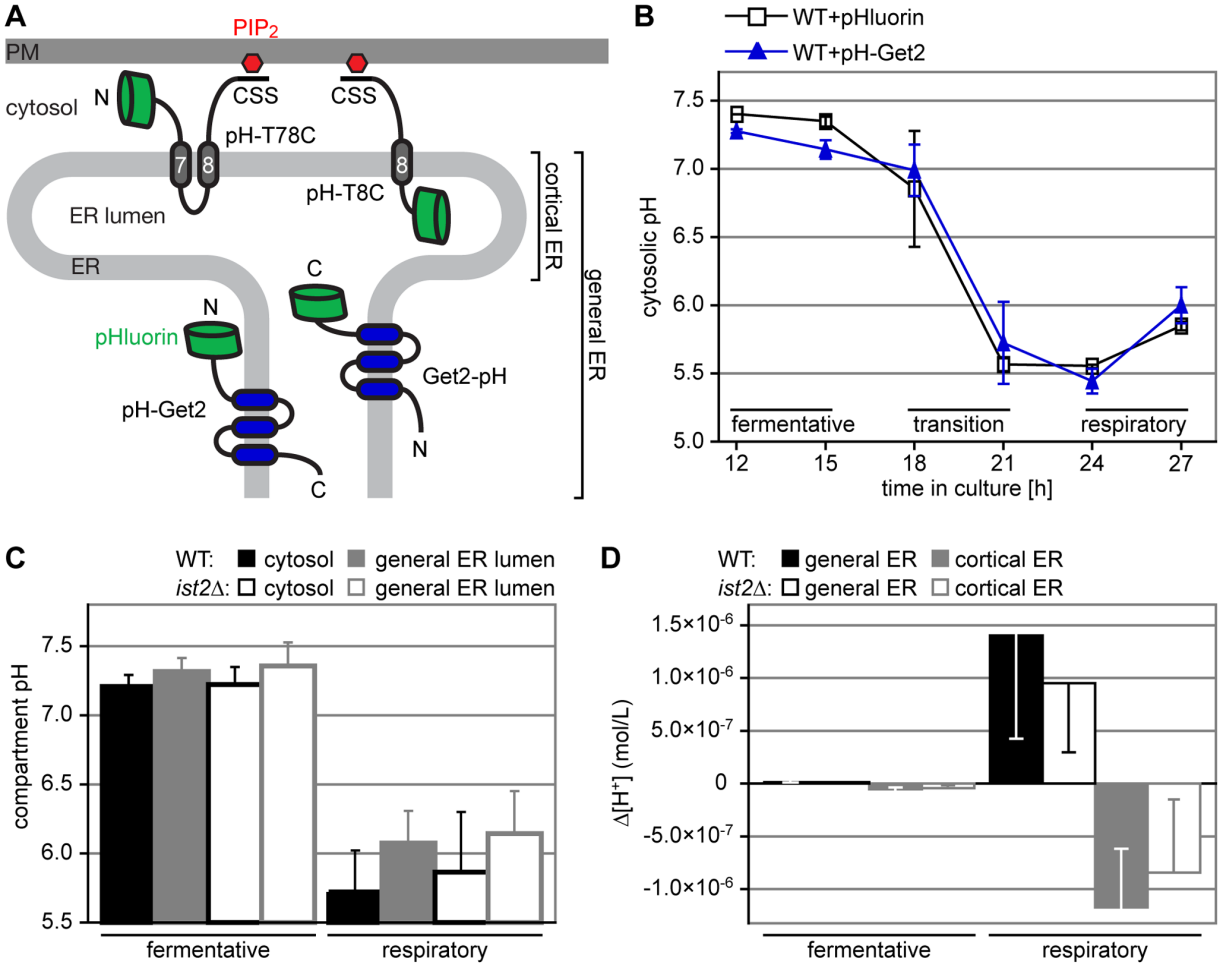
H^+^ gradients across the ER membrane depend on transition to respiration. (A) Cartoon illustrating the localization of compartment-specific pH probes. pH-T78C and pH-T8C exclusively localize to the cortical ER while Get2-pH and pH-Get2 localize to the general ER. (B) *In vivo* measurement of cytosolic pH in cells grown for 12 to 27 hours in HC medium lacking leucine. Growth phases (fermentative, transition and respiratory) are indicated. WT cells expressing pHluorin (black squares) or pH-Get2 (blue triangles) were analyzed by flow cytometry. Error bars indicate s.d.m. (n = 2). (C) pH in the cytosol (black) and general ER lumen (grey) of WT (filled bars) and *ist2*Δ (open bars) cells expressing pH-Get2 and Get2-pH under fermentative and respiratory conditions. Error bars indicate s.d.m. (n = 4). (D) H^+^ gradients (mol/L) between cytosol and lumen of the general (black) or cortical (grey) ER in WT (filled bars) and *ist2*Δ (open bars) cells under fermentative and respiratory conditions. The H^+^ concentrations of general and cortical ER cells were determined in cells expressing Get2-pH and pH-T8C, respectively. Cytosolic H^+^ concentrations in the vicinity of general and cortical ER were determined in cells expressing pH-Get2 and pH-T7C, respectively. Error bars indicate s.d.m. (n = 4).

We fused the pH-sensitive GFP derivate pHluorin, a bona fide cytosolic pH probe [25], to N- and C-termini of the ER resident membrane protein Get2. Fusion to the Get2 N-terminus generates a cytosolically exposed pH probe (pH-Get2), while fusion to the C-terminus targets the probe into the general ER lumen (Get2-pH, Fig. 2A). These probes allow the detection of potential pH gradients across the ER membrane. Colocalization with the ER marker dsRed-HDEL showed that both Get2 fusion proteins localize to the entire ER (Fig. S2A and B). Western blot analysis revealed that they were expressed similarly in WT and *ist2*Δ cells with higher expression level of the cytosolic pH-Get2 probe (Fig. S2E). Ratiometric flow cytometry with cytosolic pHluorin and pH-Get2 in fermenting WT cells revealed a slightly alkaline cytosolic pH in exponentially growing cells (Fig. 2B). Both probes indicated similar pH values of 7.2 to 7.4, suggesting that pH-Get2 faithfully reports the cytosolic pH. At the transition from fermentation to respiration between 18 and 21 hours growth the cytosolic pH decreased dramatically (Fig. 2B and [26], [27]), consistent with reduced Pma1 activity and the onset of the *ist2*Δ growth delay during this transition.

In order to measure the pH in cytosol and general ER lumen, we expressed pH-Get2 und Get2-pH in WT and *ist2*Δ cells. During fermentative growth the pH in WT and *ist2*Δ cells was alkaline and gradients across the ER membrane were not apparent (Fig. 2C). This changed when cells reached respiratory growth. The pH in the cytosol and general ER lumen acidified by 1 pH unit in both WT and *ist2*Δ cells and a pH gradient across the ER with a more acidic cytosol was established (Fig. 2C). Both cytosol and general ER lumen in WT were more acidic than in *ist2*Δ but these differences were not significant (Fig. 2C).

Since Ist2 localizes to the cortical ER, we asked whether differences in the H^+^ gradient were more pronounced between the cortical ER lumen and the cytosolic cleft located between the cortical ER and PM. In order to target pHluorin into the lumen of the cortical ER, we placed pHluorin downstream of the signal sequence of vacuolar carboxypeptidase Y and fused the protein to residues 549 to 946 of Ist2, generating pH-T8C (Fig. 2A). This sequence contains the last transmembrane domain of Ist2 and its C-terminal domain that binds PI(4,5)P_2_ at the cytosolic face of the PM. To target pHluorin into the cleft between cortical ER and PM, we placed pHluorin at the N-terminus of residues 475 to 946 of Ist2 containing the last two transmembrane domains and the C-terminal domain, generating pH-T78C (Fig. 2A). Both fusion proteins were efficiently targeted to the cortical ER (Fig. S2C and D).

H^+^ gradients across the cortical ER were restricted to cells entering respiratory growth phase, similar as seen with the Get2-based pH probes (Figs. 2C and D). Compared to the general ER, the direction of the gradient changed with higher H^+^ concentration in the cortical ER as compared to the cytosolic cleft. Differences in the H^+^ gradient between WT and *ist2*Δ were small and not significant (Fig. 2D). Thus, Ist2 had no major impact on H^+^ gradients across the ER membrane and a function of Ist2 as ion channel most likely does not explain the observed growth delay of *ist2*Δ.

**Figure 3:**
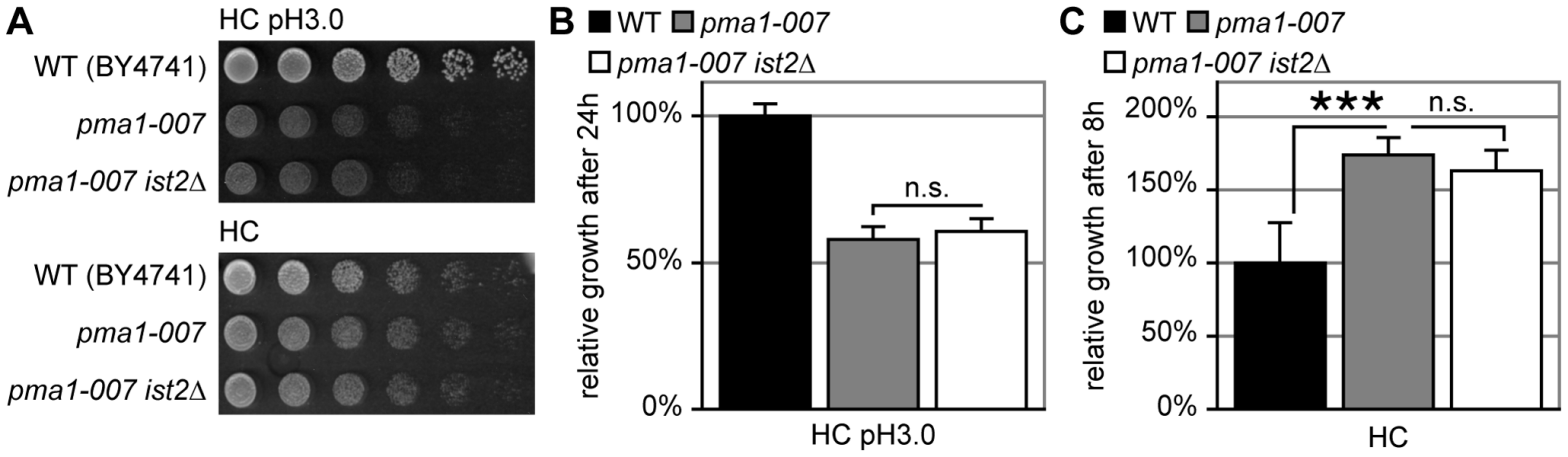
Impaired function of Pma1 does not contribute to the *ist2*Δ growth delay. (A) WT (BY4741), *pma1-007* and *pma1-007 ist2*Δ cells were grown to mid log phase and spotted on HC and HC plates buffered to pH 3.0. Cells were spotted in five-fold serial dilutions starting from 2.0 OD_600_. (B) Relative growth of WT (black), *pma1-007* (grey) and *pma1-007 ist2*Δ (white) cells. Cells were pre-cultured in HC medium for 18 hours, diluted to 0.1 OD_600_ into fresh HC medium buffered to pH 3.0 and grown for 24 hours. OD_600_ was normalized to WT. (C) As in B but cells were diluted into unbuffered HC media with a pH of 4.65 and grown for 8 hours. Error bars indicate s.d.m. (n = 9).

Since the acidification of cytosol and ER lumen coincide with the onset of the *ist2*Δ growth delay (Fig. 1D), we asked whether Pma1-generated H^+^ gradients contribute to the Ist2 phenotype. In order to test this idea, we deleted *IST2* in a *pma1-007* background, which has approximately 50% reduced Pma1 expression resulting in slow growth at pH 3.0 [28]. Compared to WT both the *pma1-007* single mutant and the *ist2Δ pma1-007* double mutant grew slow on HC plates at pH 3.0 (Fig. 3A). Importantly, the deletion of *IST2* had no impact on cell growth after dilution into media with pH 3 (Fig. 3B), suggesting that the Ist2-dependent growth defect occurs largely independently of Pma1 activity. Surprisingly, *pma1-007* single mutant and the *ist2Δ pma1-007* double mutant reached higher OD_600_ than WT after dilution into unbuffered HC media with a pH of 4.65 (Fig. 3C).

### Limited Uptake of Leucine Causes the *ist2*Δ-specific Phenotype

The *ist2*Δ-specific growth delay was restricted to cells in transition from fermentation to respiration in synthetic but not in rich media (Fig. 1B and D). Therefore, we asked whether this phenotype is related to secondary uptake of nutrients from the media, which requires specific transporters in the PM. Our WT and *ist2*Δ strains were leucine, histidine, and lysine auxotroph, which means that growth of these strains rely on leucine, histidine, and lysine uptake. HC medium contains amino acids at defined concentrations [29]. Limited availability of these amino acids together with impaired uptake may explain the *ist2*Δ growth phenotype.

**Figure 4:**
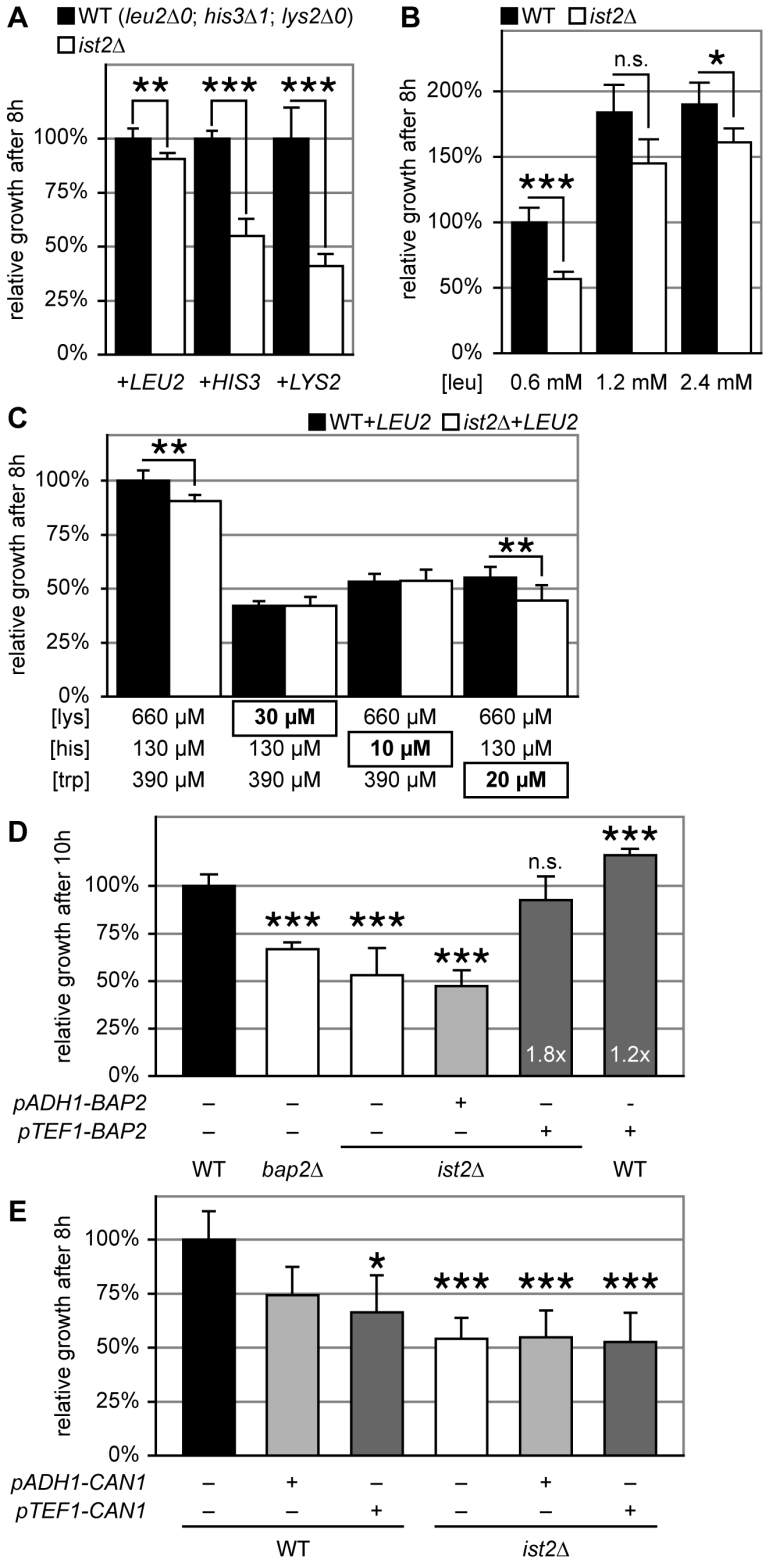
Leucine uptake is impaired in *ist2*Δ. (A) Relative growth of WT (black) and *ist2*Δ (white) strains transformed with *CEN* plasmids encoding the *LEU2*, *HIS3* or *LYS2* gene. Pre-cultures grown in HC-leu, HC-his or HC-lys media for 18 hours were diluted to 0.1 OD_600_ into fresh HC media and grown for 8 hours. OD_600_ of transformed *ist2*Δ strains were normalized to the respective WT strains. Error bars indicate s.d.m. (n ≥6). (B) Relative growth of WT and *ist2*Δ strains in HC media with the indicated leucine concentration. Note that the leu concentration of standard HC medium is 0.6 mM. Pre-cultures grown in HC medium for 18 hours were diluted to 0.1 OD_600_ into fresh media and grown for 8 hours. OD_600_ were normalized to WT grown in standard HC media. Error bars indicate s.d.m. (n = 6). (C) Relative growth of WT and *ist2*Δ strains transformed with *LEU2*-encoding *CEN* plasmids at limiting lysine (30 µM), histidine (10 µM) and tryptophane (20 µM) concentrations. Pre-cultures grown in HC-leu for 18 hours were diluted to 0.1 OD_600_ into fresh HC-leu media with the indicated lysine, histidine and tryptophane concentrations. OD_600_ were normalized to WT grown in standard HC medium. Error bars indicate s.d.m. (n ≥6). (D) Relative growth of WT, *bap2*Δ and *ist2*Δ strains transformed with *CEN* plasmids encoding *BAP2* under control of the *pADH1* (light grey) or *pTEF1* (dark grey) promoters or empty plasmids p416. Pre-cultures grown in HC-ura for 18 hours were diluted to 0.1 OD_600_ into fresh HC media and OD_600_ was measured after 10 hours. OD_600_ was normalized to WT transformed with empty plasmid. White numbers at the base of dark grey bars indicate fold increase compared to respective strains transformed with empty plasmids. Error bars indicate s.d.m. (n ≥3). (E) *CAN1* under control of *pADH1* and *pTEF1* promoters or empty vectors were expressed in WT and *ist2*Δ cells from *CEN* plasmids. Cells were grown to the end of *log* phase, diluted to 0.1 OD_600_ into fresh medium and OD_600_ was measured after 8 hours. OD_600_ was normalized to WT transformed with empty plasmid (black). Error bars indicate s.d.m. (n = 6). Significance (unpaired, two-tailed student’s t-test) of p<0.01, p<0.005 and p<0.001 is indicated by single, double and triple asterisks; n.s. indicates non-significant difference (p>0.01).

In order to test this idea, we complemented the *leu2*Δ*0*, *his3*Δ*1* and *lys2*Δ*0* deletions in WT and *ist2*Δ by transformation with *LEU2*, *HIS3* or *LYS2* containing *CEN* plasmids. Complementation of the *leu2*Δ*0* deletion rescued the *ist2*Δ growth delay, while complementation of the *his3*Δ*1* and *lys2*Δ*0* mutations did not (Fig. 4A). Consistent with a specific defect in leucine uptake, increasing the leucine concentration in the growth media from 0.6 to 1.2 mM improved growth of both *ist2*Δ and WT and reduced the growth difference between both strains (Fig. 4B). Improved WT growth is consistent with the previous observation that BY and W303 WT strains suffer from poor leucine uptake [30].

To address further whether the growth defect of *ist2*Δ is specific for the uptake of leucine, we tested growth under conditions where low concentration of lysine and histidine restrict WT growth. At 30 µM lysine and 10 µM histidine WT cells transformed with a *LEU2* plasmid reached approximately 50% of the density of cells grown in HC media containing the standard lysine and histidine concentration of 660 and 130 µM, respectively (Fig. 4C). Importantly, the deletion of *IST2* had no impact on growth under these conditions, suggesting that the *ist2*Δ-phenotype is specific for the uptake of leucine. In order to test whether deletion of *IST2* influences the uptake of other bulky and hydrophobic amino acids, we generated a tryptophan auxotroph *ist2*Δ strain. Even at 20 µM tryptophan *ist2*Δ *trp2*Δ cells grew similar to isogenic WT (Fig. 4C), suggesting that the *ist2*Δ phenotype is specific for the uptake of branched-chain amino acids.

Since increase of the leucine concentration and/or transformation with a *LEU2* plasmid rescued the growth phenotype of *ist2*Δ cells, we asked whether overexpression of the high-affinity branched-chain amino acid transporter Bap2 results in a similar rescue. We expressed *BAP2* under control of the constitutive *pADH1* and *pTEF1* promoters, because *BAP2* transcription is under control of substrate availability by the amino acid sensor complex [31]. *BAP2* overexpression under control of the stronger *pTEF1* but not under the weaker *pADH1* promoter rescued the *ist2*Δ growth phenotype to 79.8% of WT cells overexpressing *BAP2* (Fig. 4D). Similar to the increase of leucine concentration (Fig. 4B), overexpression of *BAP2* in WT improved growth 1.16 times, suggesting that leucine uptake by Bap2 restricts growth in HC media. Deletion of *BAP2* in WT background led to similar growth delay as deletion of *IST2* (Fig. 4D), suggesting that *ist2*Δ cells have an impaired expression and/or activity of Bap2. Importantly, the rescue of *ist2*Δ was specific for *BAP2* as overexpression of the arginine transporter Can1 did not rescue (Fig. 4E). On the contrary, *CAN1* overexpression delayed growth of WT cells, arguing that expression of this amino acid transporter is subject to tight control [32]. Functionality of overexpressed *CAN1* was demonstrated by a growth defect in the presence of the toxic arginine analogue canavanine (Fig. S3).

### Ist2 is Required for Bap2 Trafficking to the PM

Since the observed effects of Ist2 on H^+^-gradients across the ER were small, we analyzed whether Ist2 influences the amount of Bap2 in the PM. As a consequence of an increased distance between cortical ER and PM in *ist2*Δ [20] loss of Ist2 may increase the rate of Bap2 endocytosis. Alternatively, Ist2-mediated ER and PM coupling may generate signals affecting synthesis, stability and trafficking of Bap2 along the secretory pathway.

**Figure 5:**
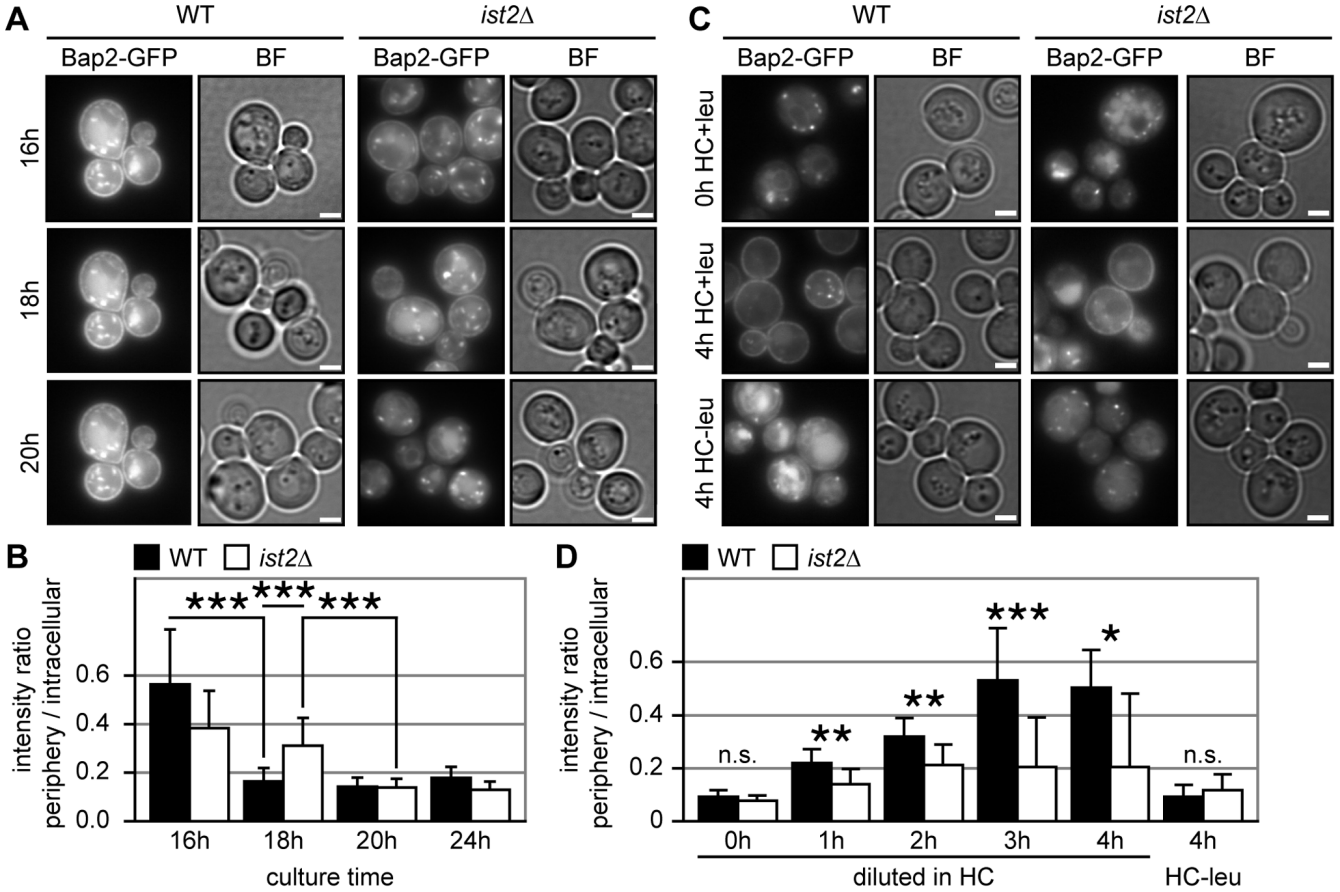
PM trafficking of Bap2 is impaired in *ist2*Δ cells. (A and C) Representative green channel (Bap2-GFP) and bright field (BF) images of WT and *ist2*Δ cells expressing genomically tagged Bap2-GFP. Cells were inoculated to 0.2 OD_600_ in HC media and grown for 16 to 20 hours (A). After 24 hours (indicated as 0 h HC+leu) cells were diluted 1∶20 into fresh HC media containing (4 h HC+leu) or lacking (4 h HC-leu) leucine and grown for 4 hours. Scale bars correspond to 2 µm. For quantification of the intensity ratio between peripheral and intracellular fluorescence the mean intensity of a ring-like peripheral area was divided by the mean intensity of the intracellular area (B and D). The quantification of the experiment shown in A includes an additional 24 hours time point (B). The quantification of the experiment shown in C includes additional time points 1 to 3 hours after dilution in HC-leu media (D). Error bars indicate s.d.m. (n = 15). Significance (unpaired, two-tailed student’s t-test) of p<0.01, p<0.005 and p<0.001 is indicated by single, double and triple asterisks; n.s. indicates non-significant difference (p>0.01).

In order to analyze the expression of Bap2, we tagged Bap2 at the C-terminus with GFP and replaced the *BAP2* with the *CYC1* 3′UTR [33]. Compared to WT expression of Bap2-GFP improved growth 2.5 times (Fig. S4A), suggesting that Bap2-GFP is functional and that fusion with GFP stabilizes the PM pool of Bap2. Epifluorescence microscopy revealed peripheral and intracellular expression of Bap2-GFP in WT and *ist2*Δ cells after 16 hours growth in HC media (Fig. 5A). At the end of the exponential phase after 18 and 20 hours growth the amount of Bap2-GFP decreased and most of the protein was removed from the PM into intracellular structures (Fig. 5A). The expression pattern of Bap2-GFP was similar in *ist2*Δ with strong decrease of peripheral signal after 20 hours. To quantify the PM pool of Bap2-GFP in WT and *ist2*Δ, we compared fluorescence intensities of the cell periphery with intracellular structures. After 16 hours the ratio between peripheral and intracellular fluorescence was 0.56±0.23 in WT and 0.38±0.15 in *ist2*Δ (Fig. 5B), suggesting a smaller Bap2 PM pool in *ist2*Δ. At later time points the ratios in WT and *ist2*Δ dropped below 0.2 (Fig. 5B), indicating a removal of most Bap2 from the PM by endocytosis at the transition from fermentation to respiration. Importantly, at this time point dilution of cultures into fresh media resulted in the most pronounced growth delay of *ist2*Δ cells, arguing that these cells suffer from insufficient amounts of Bap2 in their PMs.

Dilution of 24 hours grown cells with low amounts of Bap2-GFP, which was mostly intracellular (Fig. 5C, top row) into fresh HC media led to peripheral accumulation of Bap2-GFP (Fig. 5C, middle row). This peripheral accumulation of Bap2-GFP required the presence of leucine, suggesting that fresh media with 2% glucose and leucine trigger Bap2 synthesis and transport to the PM. Dilution into fresh leucine-free media with glucose resulted in strong intracellular accumulation of Bap2-GFP with higher expression in WT than in *ist2*Δ (Fig. 5C, bottom row). Peripheral accumulation of Bap2-GFP was faster in WT cells and they reached a ratio of periphery/intracellular signal of >0.5 after three hours (Fig. 5D). In *ist2*Δ cells this ratio increased one and two hours after dilution and remained around 0.2 (Fig. 5D). Thus, upon dilution into fresh media transport of Bap2 to the PM is less efficient in *ist2*Δ cells.

Such dramatic difference in the amount of peripheral protein was not observed in case of Can1. In order to test whether the effect of Ist2 on trafficking is specific for Bap2, we generated arginine auxotroph *ist2*Δ cells by deletion of *ARG4*, which catalyses the last step of arginine biosynthesis, and analyzed the distribution of Can1-GFP (Fig. S4B). The ratio of periphery/intracellular signal between WT, *arg4*Δ and *arg4*Δ *ist2*Δ cells were similar and differences were non-significant (Fig. S4C), arguing that expression of Can1 in the PM occurs largely independently of Ist2. Of note, the *arg4*Δ *ist2*Δ double mutant showed slow growth on HC- but not on YPD plates (Fig. S4D). Compared to the growth defect in leucine auxotroph strains, an increase of arginine concentration only improved growth of WT but had little effect on the growth of *ist2*Δ (Fig. S4E). Moreover, *CAN1* overexpression could not rescue the *arg4*Δ *ist2*Δ double mutant phenotype (Fig. 4F). Thus, the regulation of Can1 expression seems more complex and the role of Ist2 in this process remains elusive.

In order to further distinguish whether increased endocytosis or decreased trafficking of Bap2 to the PM was responsible for the observed reduction of Bap2 in the periphery of *ist2*Δ cells, we determined the compartment-specific age of Bap2 employing a tandem fluorescent protein timer (tFT) as protein tag [34]. tFT consists of a pair of fluorescent proteins that after synthesis become fluorescent with different kinetics. Superfolder (sf)GFP reaches fluorescence within minutes while slow folding mCherry maturates with a half-time of 40 min [34]. Therefore the ratios of fluorescent intensities from the two fluorescent protein domains indicate the age of a local pool of protein. To confirm that our wide-field microscopy set-up was able to report protein stability/age, we measured the fluorescent intensities of different N-degrons with an ubiquitin moiety at the N terminus. After removal of the N-terminal ubiquitin the N-degron is exposed and constructs with destabilizing N-terminal residues are marked for degradation. As shown before by ratiometric flow cytometry [34], our microscopy analysis revealed that arginine as N-degron led to fast degradation and low mCherry/sfGFP intensity ratio of 0.1±0.03, while a stabilizing threonine residue increased the mCherry/sfGFP intensity ratio to 0.59±0.18 (Fig. S5A).

**Figure 6:**
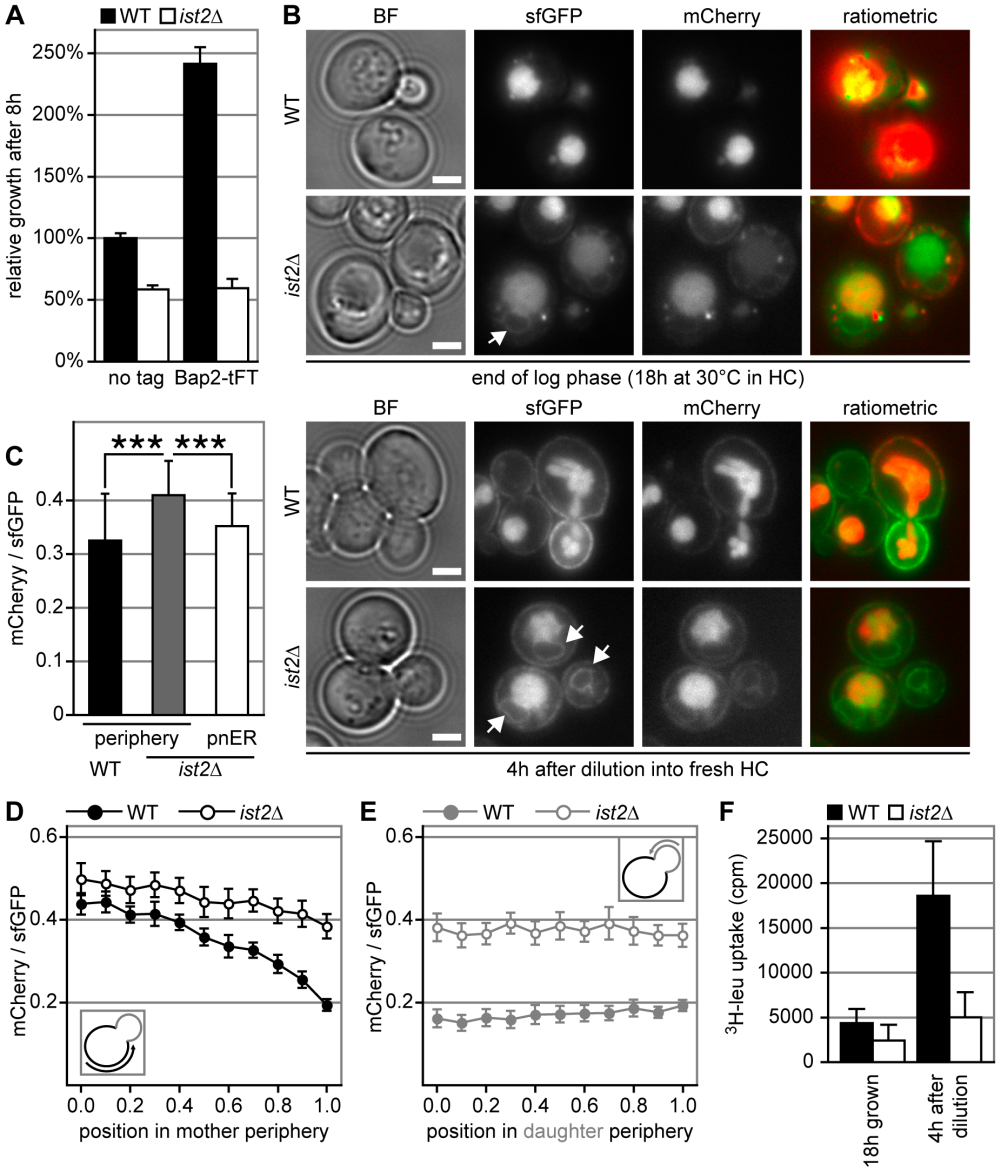
ER exit of newly synthesized Bap2 is delayed in *ist2*Δ cells. (A) Relative growth of WT (black) and *ist2*Δ (white) cells expressing endogenous Bap2 (no tag) or Bap2-tFT. Cells were grown in HC media for 18 hours, diluted to 0.1 OD_600_ into fresh medium and grown for 8 hours. OD_600_ was normalized to WT expressing untagged Bap2. Error bars indicate s.d.m. (n = 6 for untagged and n = 15 for tFT-tagged cells). (B) Representative images of Bap2-tFT expressed in WT and *ist2*Δ cells. Bright field (BF), green channel (sfGFP), red channel (mCherry) and ratiometric images are shown after 18 hours growth in HC media or 4 hours after dilution into fresh HC media. Arrows in sfGFP images indicate perinuclear ER. Scale bars in BF images correspond to 2 µm. (C) mCherry/sfGFP intensity ratios of the periphery in WT and *ist2*Δ cells and of perinuclear ER in *ist2*Δ. Error bars indicate s.d.m. (n = 40; 10 cells each from 4 images taken with identical settings. Single cell values correspond to mean intensity of three randomly chosen areas within the respective membrane). (D and E) Distribution of the mCherry/sfGFP intensity ratio for Bap2-tFT in the periphery of WT (filled circles) and *ist2*Δ (open circles) mother (D) and daughter (E) cells. Error bars indicate s.e.m. (n ≥17). (F) Uptake of ^3^H-leucine in WT and *ist2Δ* cells expressing genomically tagged Bap2-tFT and *LEU2* from a plasmid. Cells were grown for 18 hours in HC media without leucine followed by dilution into HC media containing leucine.

We tagged *BAP2* in WT and *ist2*Δ at its C-terminus with the tFT tag. This improved growth of WT (Fig. 6A), suggesting that Bap2-tFT is a functional leucine transporter. Moreover, the mCherry/sfGFP moiety at the C terminus may interfere with endocytosis, which would stabilize the PM pool of Bap2-tFT. Comparable improvement of growth was seen for expression of Bap2-GFP in WT (Fig. S4A). In contrast to WT, expression of Bap2-tFT in *ist2*Δ had no positive effect on growth and the strain maintained the *ist2*Δ growth delay phenotype (Fig. 6A), indicating that Ist2 is required for Bap2 function.

After 20 hours growth in HC media, most of the Bap2-tFT accumulated in vacuoles of WT, while *ist2*Δ cells showed additional dot-like intracellular signal as well as signal in the cell periphery and in ring-like structures (Fig. 6B). The ring-like structures correspond to the perinuclear ER (Fig. 6B, indicated by arrows) and had a brighter sfGFP signal compared to the mCherry signal, indicating accumulation of de novo synthesized Bap2-tFT in the ER of *ist2*Δ.

Dilution into fresh HC media led to peripheral accumulation of Bap2-tFT in WT cells with pronounced accumulation of peripheral sfGFP signal in daughter cells (Fig. 6B), suggesting that newly synthesized Bap2-tFT traveled along the polarized secretory pathway into the PM of daughters, while *ist2*Δ cells had less Bap2-tFT in the periphery and accumulated Bap2-tFT in the perinuclear ER. A comparison between the peripheral mCherry/sfGFP intensity ratios of WT and *ist2*Δ cells revealed slightly older Bap2-tFT in *ist2*Δ as compared to WT (Fig. 6C). This argues against accelerated Bap2 endocytosis in *ist2*Δ. This interpretation however remains difficult, since the limited spatial resolution of light microscopy does not allow to distinguish whether the peripheral signal corresponds to Bap2-tFT in the cortical ER or PM. Quantification of mCherry/sfGFP intensity ratios in different regions of the periphery of mother cells, showed a high ratio of 0.44±0.03 to 0.5±0.04 at the distal mother cell tip in WT and *ist2*Δ (Fig. 6D), suggesting an accumulation of old Bap2-tFT in distal regions of the PM. Close to the bud neck the mCherry/sfGFP intensity ratios were 0.19±0.02 in WT and 0.38±0.03 in *ist2*Δ cells, suggesting transport of young Bap2-tFT towards domains of the PM close to the bud neck in WT and delayed transport to these regions of the PM in *ist2*Δ. Consistent with a delayed transport of Bap2 to expanding regions of the PM in *ist2*Δ, the periphery of WT daughter cells had mCherry/sfGFP intensity ratios below 0.2 indicating young Bap2-tFT, while this ratio was almost twice as high in *ist2*Δ daughter cells (Fig. 6E).

In order to compare Bap2 activity in WT and *ist2*Δ cells, we measured uptake of ^3^H-leucine. Therefore, cells expressing *LEU2* from a plasmid were grown for 18 hours in HC media without leucine and Bap2-tFT expression was induced by dilution into leucine containing HC media. Before dilution Bap2-tFT was depleted from the PM (Fig. S5B) and both WT and *ist2*Δ showed low ^3^H-leucine uptake (Fig. 6F). Four hours after dilution Bap2-tFT reached the PM, while many *ist2*Δ cells showed additional perinuclear ER signal (Fig. S5B). This correlated with 4.3 fold increase of ^3^H-leucine uptake in WT compared to 2.1 fold increase in *ist2*Δ (Fig. 6F), suggesting that reduced expression of Bap2 in the PM of *ist2*Δ explains the observed growth defect.

The perinuclear ER signal in diluted *ist2*Δ cells had a mCherry/sfGFP intensity ratio of 0.35±0.06 (Fig. 6C) and two hours after dilution 80% of *ist2*Δ cells accumulated Bap2-tFT in the perinuclear ER, while this ER accumulation was observed only in a few WT cells (Fig. S5C). The mCherry/sfGFP intensity ratios of the perinuclear ER in *ist2*Δ and the entire cell periphery in WT were similar, suggesting that Bap2-tFT spends a relatively long time in the perinuclear ER of *ist2*Δ cells before the protein travels to the PM. Taken together, efficient trafficking of Bap2 from perinuclear ER to the PM requires Ist2 and this growth phase-specific delay in Bap2 trafficking correlates with the leucine-dependent growth phenotype of *ist2*Δ cells.

## Discussion

Ist2 in the cortical ER is required for efficient transport of newly synthesized Bap2 to the PM. This is relevant for growth of yeast cells under changing environmental conditions, i.e. during the transition from fermentation to respiration. The amount of Bap2 in the PM is low at the end of fermentation and rapid growth of leucine auxotroph strains depends on new synthesis and transport of Bap2 from the ER to the PM. Degradation of Bap2 and other amino acid transporters in cultures reaching the end of fermentation is a common phenomenon and had been observed for tryptophan, lysine and arginine transporters Tat2, Lyp1 and Can1, respectively [3], [35]–[38]. Since the deletion of *IST2* in tryptophan, lysine and histidine auxotroph cells had no effect on growth under conditions with limiting supply of these amino acids, the effect of Ist2 on trafficking of Bap2 seems rather specific.

Consistent with previous studies, we found a marked drop of cytosolic pH at the end of fermentative growth [26], [39], [40]. This can be explained with a decrease of Pma1 activity and down regulation of *PMA1* expression upon glucose starvation [22], [41], [42] and results in a decreased H^+^ gradient across the PM. Compromised Pma1 activity at the end of fermentation in combination with low Bap2 PM expression in *ist2*Δ explain the growth delay under conditions that require leucine uptake. Consistently, respiratory conditions with higher Pma1 activity [43] reduced the *ist2*Δ growth delay. However, low Pma1 activity alone had no effect on growth of *ist2*Δ and promoted adaptation from diauxic shift to fermentation in *pma1-007*. Ist2-mediated coupling may contribute to down-regulation of Pma1 indirectly by providing an inter-membrane signaling platform [44]. Consistent with a role of Ist2 in signaling, we previously reported a negative genetic interaction with the casein kinase I Yck1 that down-regulates Pma1 activity in response to glucose starvation [20], [42].

Given that the ER membrane is permeable to H^+^ and the ER lumen pH equilibrates with the cytosol [45], one would expect no gradients across this compartment. However, V-ATPase, that acidifies compartments along the secretory pathway [46], was suggested also to function in the ER [47]. Active transport of H^+^ into the ER lumen would allow ER acidification if H^+^ do not diffuse freely across the ER membrane. By coupling of pHluorin to the N- and C-terminus of an integral ER membrane protein with uneven number of transmembrane domains, we could detect H^+^ gradients that occurred specifically after depletion of glucose when cells switched from fermentation to respiration. These gradients showed subcompartment-specific differences with a more acidic cortical ER compared to the cytosol. This is consistent with the idea of differences in composition and function of individual ER domains, e.g. with thin cortical ER in mammalian cells that is free of the abundant ER-resident heat shock homologue BIP and functions in store-operated Ca^2+^ entry [48].

Although, expression of our luminal subcompartment-specific probes was low, ratiometric measurements by flow cytometry led to a sufficient signal to noise ratio. Compared to the previously used plate reader assay the sensitivity of this assay was much better [20], [49]–[51] and therefore appropriate for the detection of H^+^-gradients across the ER. Difficulties in the comparison of H^+^ gradients between WT and *ist2*Δ arose from growth differences and growth phase-dependent formation of H^+^-gradients as well as from the induction of additional ER-PM contacts upon expression of the cortical pH probes. Since the observed differences in H^+^-gradients between WT and *ist2*Δ were small and within the experimental error, these data do not support an ion channel function of Ist2 as seen for some of its counterparts in the mammalian PM [14]–[17].

The observed toxicity of Can1 overexpression indicated that yeast cells regulate the abundance and function of Can1 transporter precisely. This is consistent with regulation of cytosolic arginine concentration, e.g. by Btn1-mediated arginine transport into the vacuole [52]. How Ist2 influences arginine homeostasis remains unkown but a previously observed strain-specific growth defect of *btn1Δ ist2*Δ double mutants supports a role of Ist2 in arginine homeostasis [53].

Increased Bap2 expression, either by C-terminal tagging or by overexpression of untagged protein rescued the Ist2-specific growth delay. The positive effect by tagging suggests that reduced endocytosis in *ist2*Δ improves PM expression. The tag may mask endocytosis signals and prevent palmitoylation at a C-terminal FWC motif, which was suggested to regulate protein trafficking and function [54]. Reduced coupling of cortical ER and PM and improved access of the endocytosis machinery as consequence of *IST2* deletion may enhance endocytosis rate [55]. However, this seemed rather specific for Bap2. Increased age of Bap2-tFT in the PM of *ist2*Δ cells argue against rapid endocytosis as only source of the growth delay phenotype and improved growth by Bap2 tagging required Ist2 function, consistent with a role of Ist2 for forward trafficking of Bap2.

Using tFT as protein tag [34], we were able to follow the fate of Bap2 along the secretory pathway and obtained information about compartment-specific age of the protein. As described earlier for the hexose transporter Hxt1 and Pma1 [34], we observed accumulation of young Bap2-tFT in growing daughter cells and we resolved domain-specific difference in protein age. This makes tFT a powerful tool for studying diverse aspects of protein trafficking.

Exit of multiple amino acid transporters from the ER depends on the ER chaperone Shr3, while export of other PM proteins occurs independently of Shr3 [56], [57]. Thus ER exit of amino acid transporters is a demanding step for the cellular quality control system. Delayed exit of Bap2 in *ist2*Δ cells and probably of other proteins may explain constitutively active unfolded protein response pathway in Δtether mutants, where all proteins involved in ER-PM contact formation, including Ist2, were deleted [9]. Whether defects in ER morphology caused the perinuclear ER Bap2 accumulation or whether loss of Ist2 causes other defects remains open. Cortical localization of Ist2 and perinuclear accumulation of Bap2-tFT argues against a direct effect of aberrant ER morphology. We favor a role of Ist2 in trans-signaling between cortical ER and PM. Candidates for dys-regulated signals are phosphoinositide lipids, which function as important regulators of intracellular trafficking [58], [59]. The ER-resident lipid phosphatase Sac1 converts PI(4)P at the cytosolic side of the PM into PI and this activity requires ER-PM contacts [11]. Complex formation between Ist2 and Sac1 [9] and Ist2-mediated recruitment of cortical ER to the PM [20] support a role of Ist2 in the formation of inter-membrane signaling platforms. In addition to a membrane tethering function, small Ist2-dependent changes in H^+^ flux across the cortical ER may serve as signal for Bap2 trafficking as cytosolic pH has been described as signal for proteasome homeostasis, mitochondrial function, chronological ageing and nutrient transporter trafficking [60]–[62].

In summary, we found that Ist2 in the cortical ER is required to cope with environmental changes through equipment of the PM with specific transporters. Efficient ER export of the leucine transporter Bap2 to the PM depends on Ist2 and involves as yet unknown communication between ER and PM.

## Materials and Methods

### Yeast Strains

All yeast strains used were descendents of BY4741/42 [63]. The *ist2*Δ strain (MSY399) was previously described [20]. The *IST2* gene was deleted by chromosomal integration of PCR amplified natNT2 in *pma1-007*, *trp2*Δ and *arg4*Δ strains or kanMX4 in BY4742 [33]. Gene deletions were validated by PCR. The *CAN1* and *BAP2* genes were C-terminally tagged in BY4742 and MSY399 [33] resulting in MSY668 (WT, *CAN1-GFP*), MSY669 (*ist2*Δ, *CAN1*-*GFP*), MSY670 (WT, *BAP2*-*GFP*), and MSY673 (*ist2*Δ, *BAP2*-*GFP*). Strains expressing Ubi-R-tFT, Ubi-E-tFT, Ubi-I-tFT, Ubi-M-tFT and Ubi-T-tFT were previously described [34]. BY4742 WT and *ist2*Δ strains expressing *BAP2* with the C-terminal tFT tag (mCherry-sfGFP) were generated using a seamless tagging strategy [64].

### Media and Growth Conditions

HC media contained 20 mg/L adenine, 35 mg/L uracil, 20 mg/L arginine, 100 mg/L aspartic acid, 100 mg/L glutamic acid, 20 mg/L histidine, 80 mg/L isoleucine, 80 mg/L leucine, 120 mg/L lysine, 20 mg/L methionine, 50 mg/L phenylalanine, 400 mg/L serine, 200 mg/L threonine, 80 mg/L tryptophan, 60 mg/L tyrosine, 150 mg/L valine (all amino acids from Sigma-Aldrich), 6.7 g/L Difco Yeast nitrogen base (Becton Dickinson) and 2% glucose [29]. For physiological experiments yeast cells were grown at 30°C for the indicated times in 5 mL of the indicated media in glass tubes on a rolling wheel. The pH of YPD plates was buffered with 50 mM 2-(N-morpholino)ethanesulfonic acid (MES) and adjusted with HCl. The glucose concentration of yeast growth media cleared of cells by centrifugation (15,000 g, 1 min at room temperature) was determined using a D-glucose test kit (R-Biopharm) enzymatically converting glucose to NADPH. Media samples were diluted 1∶10, 1∶20 or 1∶40 in water for glucose concentrations >0.5 g/L, >9.0 g/L or >14.0 g/L, respectively. NADPH concentration was determined by absorbtion at 365 nm against water in a spectrophotometer (Ultrospec3000, Pharmacia Biotech).

### Plasmids

Ratiometric pHluorin probes were encoded by p415 plasmids containing the *TEF1* 5′ UTR and *CYC1* 3′ UTR [65]. pMS588, encoding cytosolical pHluorin was previously described [20]. pMS608, encoding pHluorin fused to the C-terminus of *GET2* via a NotI restriction site was generated by Blanche Schwappach (Department of Biochemistry I, Universitätsmedizin Göttingen and Max-Planck-Institut für biophysikalische Chemie, Göttingen, Germany). pHluorin was fused to the N-terminus of *GET2* via a SacII restriction site, resulting in pMS634. Nucleotides 1413 to 2838 and 1191 to 2838 of *IST2* were fused C-terminally to pHluorin using SacII and NotI restriction sites resulting in pMS635 and pMS643, respectively. In pMS643 pHluorin was N-terminally fused to the signal sequence of *PRC1*. pMS650 encoding GFP-*IST2* under control of endogenous *IST2* UTRs was produced from pMS602 [20] by introducing a ClaI restriction site into the *HIS3* gene. *CAN1* and *BAP2* were introduced into p416 plasmids containing the *ADH1* or *TEF1* 5′ UTR and *CYC1* 3′ UTRs [65] resulting in pMS724 (*pADH*-*CAN1*) and pMS725 (*pTEF1*-*CAN1*) using XmaI and XhoI restriction sites and pMS757 (*pADH1*-*BAP2*) and pMS758 (*pTEF1*-*BAP2*) using XmaI and SalI restriction sites, respectively.

### pH Measurements

For measurement of intracellular pH by flow cytometry (FACScanto, Becton Dickinson), cells were grown in HC media at 30°C and diluted immediately before the measurement to <0.5 OD_600_ into medium of the same culture cleared from cells by centrifugation. After excitation at 405 and 488 nm, fluorescence emission was sequentially filtered using 502 nm long-pass and 530/30 nm and 510/50 nm band-pass filters, respectively. Fluorescence was corrected for background by gating out cells transformed with empty plasmid (p415). The ratio of mean fluorescence (n >3500 cells) after excitation with 405 nm and 488 nm was calculated. Data were processed using FACSDiva (Becton Dickinson) and Microsoft Excel (Microsoft corporation) software. Cytosolic and ER luminal pHluorin were calibrated with pH using calibration curves generated by permeabilization of cells with 0.16% digitonin (Calbiochem, High Purity, #300410) in HC media buffered to pH 6.2, 6.4, 6.6, 6.8, 7.0, 7.2 and 7.4 with 100 mM 3-(N-morpholino)-propanesulfonic acid (MOPS).

### Generation of Yeast Cell Lysates and Western Blotting

Yeast whole cell lysates were generated by alkaline membrane lysis (15 min incubation on ice in 150 µL 1.85 M NaOH, 7.5% β-mercaptoethanol) and protein denaturation (≥10 min incubation on ice after addition of 150 µL 55% trichloroacetic acid and 1 mL water). Denatured proteins were pelleted at 4°C and 18000 g for 10 min. Pellets were washed with 150 µL 20 mM Tris (pH 7.4), 80% acetone, resuspended in HU-buffer (8 M urea, 5% SDS, 200 mM Tris–HCl, pH 6.8, 1 mM EDTA, 0.05% bromphenol blue, 4% β-mercaptoethanol), incubated for 30 min at 37°C, and separated by 7.5% SDS–PAGE followed by Western blotting using GFP-specifc antibodies (1∶20,000, rabbit serum, gift from Dirk Görlich, Max-Planck-Institute for Biophysical Chemistry, Göttingen, Germany). Detection and quantification of protein bands from Western blots was done using the LAS-1000 system (Fujifilm) followed by image processing using MultiGauge (Fujifilm) and ImageJ software.

### Epifluorescence Microscopy and Image Processing

Epifluorescence microscopy was performed as described before [20]. Strains expressing Bap2-tFT were imaged with identical settings for corresponding samples using a total internal reflection fluorescence microscope and laser illumination as described [20]. Images were processed using ImageJ (http://rsb.info.nih.gov/ij/). Images of strains expressing Bap2-tFT were flatfield corrected. Ratiometric images were processed by GFP-intensitiy weighted mCherry/GFP ratiometric image colorisation as described before [34]. For presentation of individual cells, background intensity was subtracted from raw images and brightness and contrast was optimized. For quantification of the ratio between peripheral and intracellular fluorescence, regions-of-interest were applied to the cell periphery, which was defined by two circles surrounding the most peripheral signal with average distance of 500 nm between both circles. Signal inside of the inner circle was counted as intracellular. For quantification of mCherry/sfGFP intensity ratios in different regions of the periphery of mother and daugther cells (Fig. 5D and E) the signal intensities ratios along the cell peripheries were measured and plotted against relative distance from both distal poles (0.0) towards the bud neck (1.0).

### Leucine Uptake Assay

Leucine uptake of yeast cells was measured by quantification of ^3^H-leucine incorporation. Cells transformed with a *LEU2* plasmid were grown in HC media without leucine for 18 hours followed by dilution into fresh HC media containing leucine, washed twice with HC media without leucine and sedimented by centrifugation (16200 g for 2 min at room temperature). Pellets were resuspended in 50 µL HC medium containing 6 µM leucine and 0.6 µCi ^3^H-leucine (PerkinElmer, NET135H250UC) and incubated for 15 min at 30°C. Cells were washed twice with icecold HC media without leucine and resuspended in 3 mL scintillation liquid (Ultima Gold, PerkinElmer). Radioactivity (counts per min) was measured in a scintillation counter (Beckman LS6000IC).

## Supporting Information

Figure S1 **Absolute growth of WT and** ***ist2*** **Δ cells.** (A) WT and *ist2*Δ cells were grown in HC media for 18 hours at 30°C and single cells were isolated on HC (n = 100) and YPD (n = 50) plates using a dissection microscope. These plates were incubated for 5 days at 30°C and formation of colonies was classified as “not formed” (white) and “large” (black). Colonies which had up to half of the diameter of the majority of large colonies were classified as “small” (grey). (B) WT (closed squares) and *ist2*Δ (open squares) cells were grown in HC media at 30°C. Cells were diluted to 0.1 OD_600_ from pre-cultures grown in HC media for 18 hours at 30°C. (C) OD_600_ of WT (black) and *ist2*Δ (white) cells 8 hours after dilution to 0.1 OD_600_ into fresh HC media from cells in Fig. 1D. Error bars indicate s.d.m. (n = 3).
(TIF)

Figure S2 **Validation of compartment-specific pH-probes.** (A–D) Epifluorescence microscopy images of WT cells expressing the luminal ER marker dsRed-HDEL with either pH-Get2 (A), Get2-pH (B), pH-T78C (C) or pH-T8C (D). Upper row shows green (pHluorin) and lower row red (dsRed-HDEL) channel. Scale bar corresponds to 2 µm. (E) Western blot of whole cells lysates from WT and *ist2*Δ cells expressing empty p415 plasmid, Get2-pH or pH-Get2. Nitrocellulose membrane was stained with PonceauS for loading control.
(TIF)

Figure S3 **Arginine homeostasis depends on Ist2.** WT and *can1*Δ cells transformed with empty p416 plasmids or plasmids encoding *CAN1* under control of the *pADH1* or *pTEF1* promoter. Cells were spotted on HC plates with 1 mg/L canavanine or HC-ura plates.
(TIF)

Figure S4 **Can1-GFP trafficking does not depend on Ist2.** (A) Relative growth of WT (black), *ist2*Δ (white), *bap2*Δ (grey) and WT cells expressing genomically tagged Bap2-GFP (green) in HC media. Cells were grown for 18 hours, diluted to 0.1 OD_600_ into fresh media and grown for 8 hours. OD_600_ was normalized to WT. Error bars indicate s.d.m. (n ≥6). (B) Representative epifluorescence images of WT, *arg4*Δ and *arg4*Δ *ist2*Δ cells expressing genomically tagged Can1-GFP. Cells were inoculated to 0.2 OD_600_ in HC media and grown for 24 hours (indicated as 0 h), diluted 1∶20 into fresh HC media containing 1.2 mM leucine and grown for 4 hours. Scale bar corresponds to 2 µm. (C) Quantification of the peripheral to intracellular Can1-GFP fluorescence intensity ratio in cells presented in B. Error bars indicate s.d.m. (n = 15). (D) WT, *ist2*Δ, *arg4*Δ, *arg4*Δ *ist2*Δ, and *arg4*Δ *ist2*Δ cells expressing *GFP*-*IST2* under control of the endogenous *IST2* promoter from the *his3*Δ*1* locus were spotted on YPD and HC media in five-fold serial dilutions starting from 2 OD_600_ and grown for 3 days at 25°C. (E) Relative growth of *arg4*Δ (black) and *arg4*Δ *ist2*Δ (white) cells in HC media with the indicated arginine concentrations. Note that the arginine concentration of standard HC medium is 95 µM. Pre-cultures grown in YPD medium for 18 hours were diluted to 0.05 OD_600_ into HC medium with the indicated arginine concentrations and grown for 16 hours. OD_600_ were normalized to WT grown in HC containing 95 µM arginine. Error bars indicate s.d.m. (n = 6). (F) *CAN1* under control of *pADH1* and *pTEF1* promoters or empty plasmids were expressed in *arg4*Δ and *arg4*Δ *ist2*Δ cells from *CEN* plasmids. OD_600_ was normalized to *arg4*Δ transformed with empty plasmid. Error bars indicate s.d.m. (n = 12). Significance (unpaired, two-tailed student’s t-test) of p<0.005 and p<0.001 is indicated by double and triple asterisks.
(TIF)

Figure S5 **Bap2 accumulates in the perinuclear ER of** ***ist2***Δ**.** (A) mCherry/sfGFP fluorescence intensity ratios in cells expressing the indicated Ubi-X-mCherry-sfGFP constructs measured by epifluorescence microscopy. Mean mCherry and sfGFP intensities of a cytoplasmic region with a diameter of 537 pixels were quantified and are shown as mean+s.d.m (n = 100, except Ubi-I n = 69). Values for each construct are significantly different from neighbouring ones (p<0.001; unpaired, two-tailed student’s t-test). (B) Representative bright field (BF) and sfGFP images of Bap2-tFT expressed in WT and *ist2*Δ cells transformed with a *LEU2* plasmid. Cells were grown for 18 hours in HC media without leucine followed by dilution into HC media containing leucine. Arrows in sfGFP images indicate perinuclear ER and scale bar corresponds to 2 µm. (C) Quantification of perinuclear ER Bap2-tFT signals (sfGFP channel) in WT and *ist2*Δ cells at different timepoints after dilution into fresh HC medium from 18 hours grown pre-cultures. Mean of four images with ≥12 cells chosen from bright field channel is shown. Error bars indicate s.d.m. and triple asterisks indicate significant difference (p<0.001; unpaired, two-tailed student’s t-test).
(TIF)
