# Supplementary figures and images for: Ist2 in the Yeast Cortical Endoplasmic Reticulum Promotes Trafficking of the Amino Acid Transporter Bap2 to the Plasma Membrane
Source: PLoS One. 2014 Jan 8;9(1):e85418. doi: 10.1371/journal.pone.0085418 (PMC3885692; doi:10.1371/journal.pone.0085418)

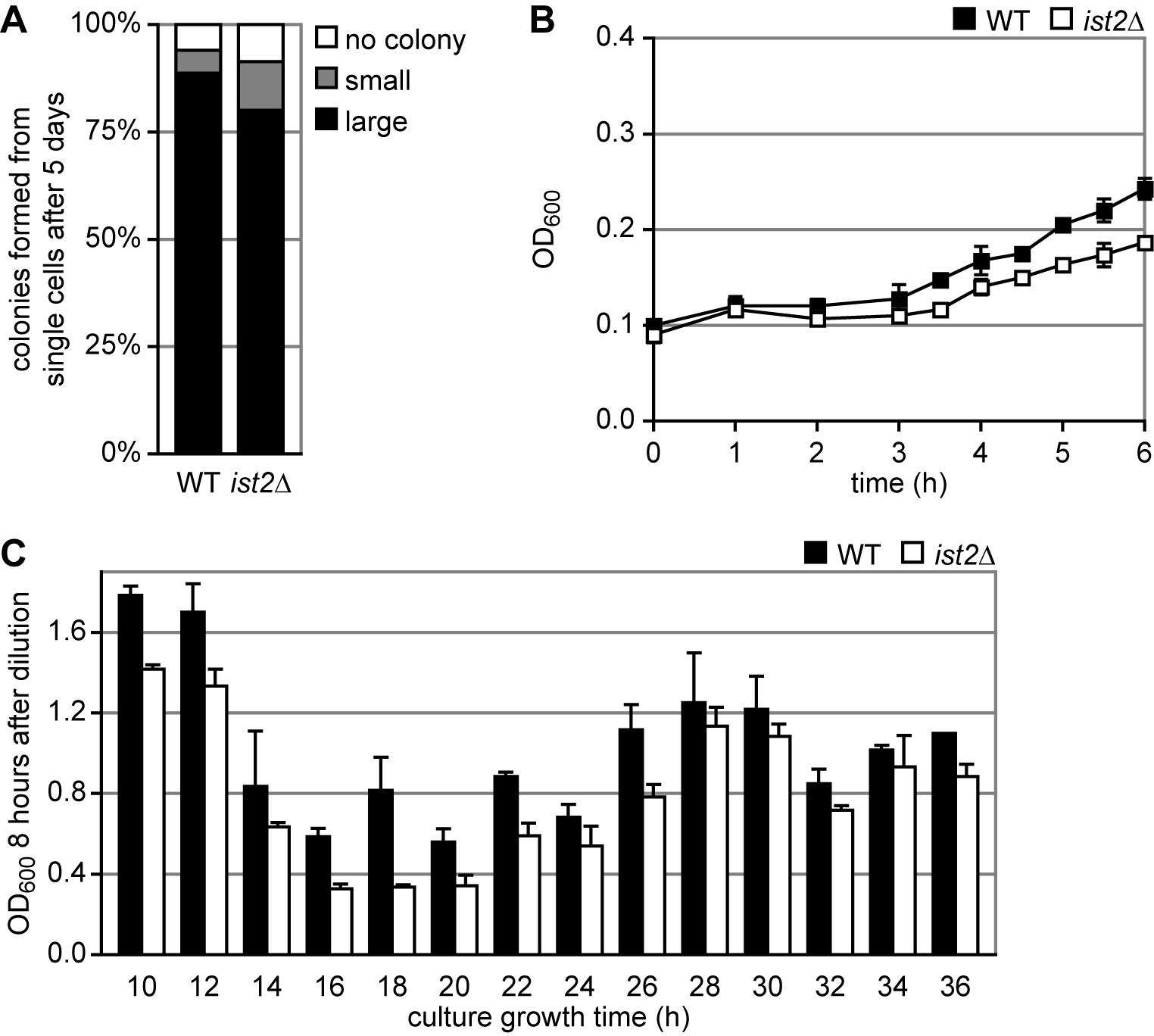

Supplement: Figure S1 — Absolute growth of WT and ist2 Δ cells. (A) WT and ist2Δ cells were grown in HC media for 18 hours at 30°C and single cells were isolated on HC (n = 100) and YPD (n = 50) plates using a dissection microscope. These plates were incubated for 5 days at 30°C and formation of colonies was classified as “not formed” (white) and “large” (black). Colonies which had up to half of the diameter of the majority of large colonies were classified as “small” (grey). (B) WT (closed squares) and ist2Δ (open squares) cells were grown in HC media at 30°C. Cells were diluted to 0.1 OD600 from pre-cultures grown in HC media for 18 hours at 30°C. (C) OD600 of WT (black) and ist2Δ (white) cells 8 hours after dilution to 0.1 OD600 into fresh HC media from cells in Fig. 1D. Error bars indicate s.d.m. (n = 3). (TIF) [file pone.0085418.s001.tif]

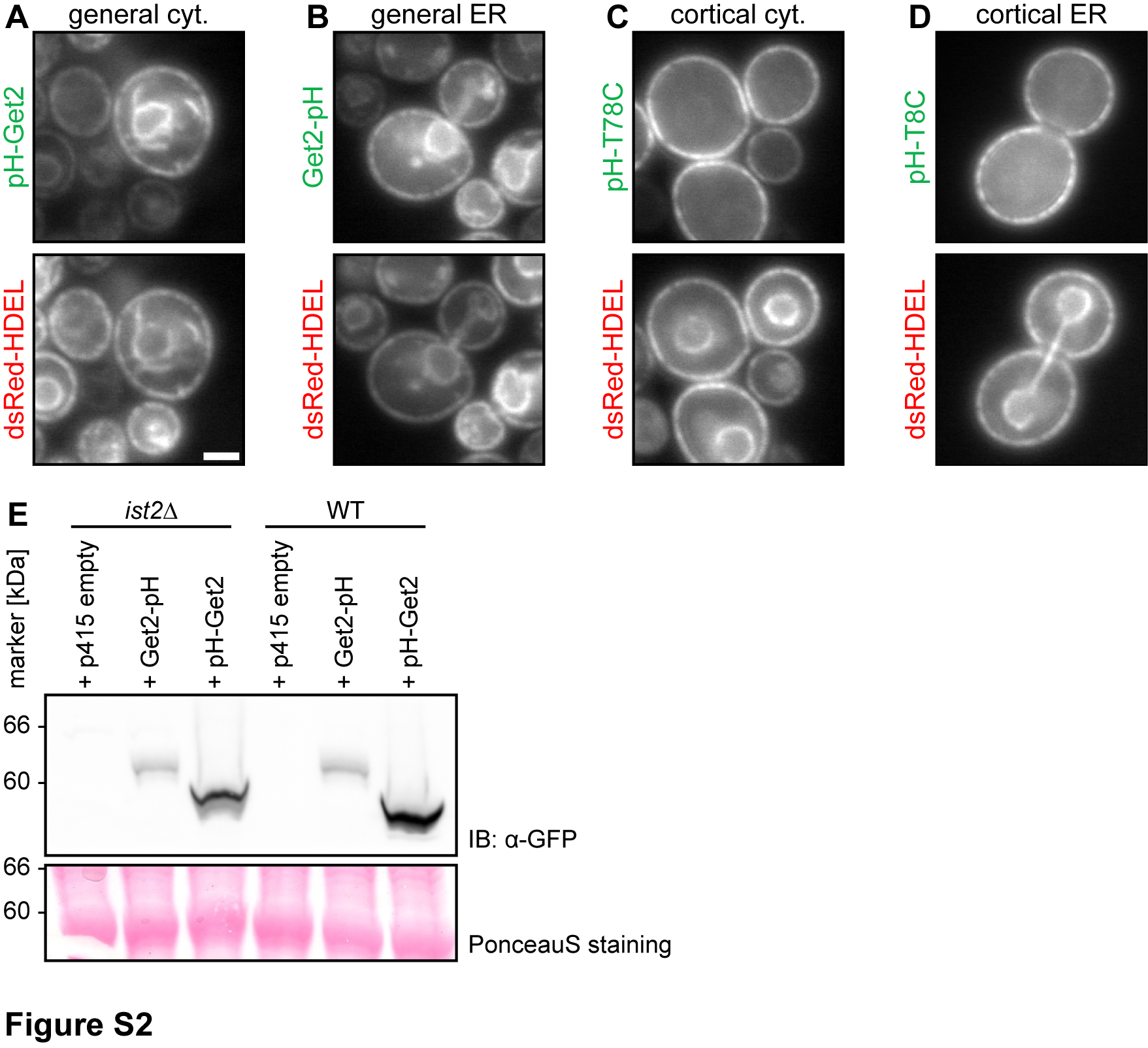

Supplement: Figure S2 — Validation of compartment-specific pH-probes. (A–D) Epifluorescence microscopy images of WT cells expressing the luminal ER marker dsRed-HDEL with either pH-Get2 (A), Get2-pH (B), pH-T78C (C) or pH-T8C (D). Upper row shows green (pHluorin) and lower row red (dsRed-HDEL) channel. Scale bar corresponds to 2 µm. (E) Western blot of whole cells lysates from WT and ist2Δ cells expressing empty p415 plasmid, Get2-pH or pH-Get2. Nitrocellulose membrane was stained with PonceauS for loading control. (TIF) [file pone.0085418.s002.tif]

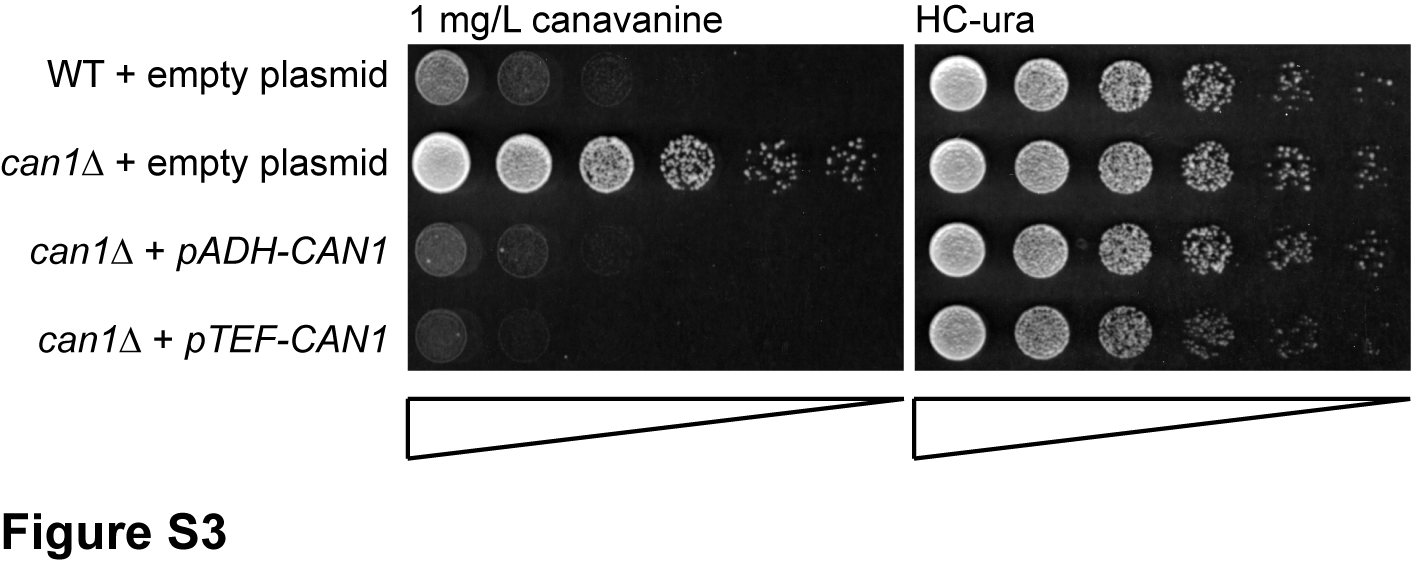

Supplement: Figure S3 — Arginine homeostasis depends on Ist2. WT and can1Δ cells transformed with empty p416 plasmids or plasmids encoding CAN1 under control of the pADH1 or pTEF1 promoter. Cells were spotted on HC plates with 1 mg/L canavanine or HC-ura plates. (TIF) [file pone.0085418.s003.tif]

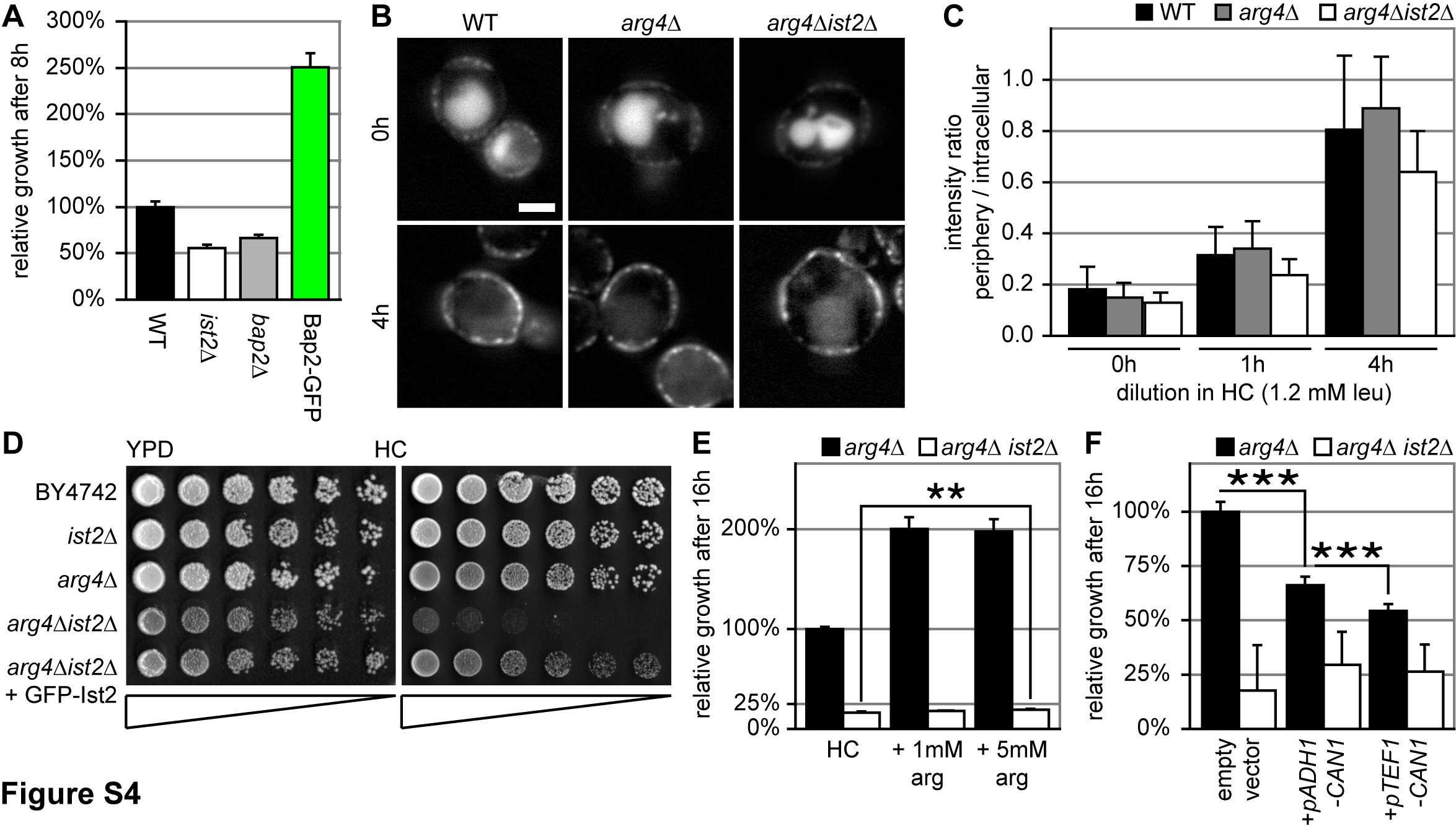

Supplement: Figure S4 — Can1-GFP trafficking does not depend on Ist2. (A) Relative growth of WT (black), ist2Δ (white), bap2Δ (grey) and WT cells expressing genomically tagged Bap2-GFP (green) in HC media. Cells were grown for 18 hours, diluted to 0.1 OD600 into fresh media and grown for 8 hours. OD600 was normalized to WT. Error bars indicate s.d.m. (n ≥6). (B) Representative epifluorescence images of WT, arg4Δ and arg4Δ ist2Δ cells expressing genomically tagged Can1-GFP. Cells were inoculated to 0.2 OD600 in HC media and grown for 24 hours (indicated as 0 h), diluted 1∶20 into fresh HC media containing 1.2 mM leucine and grown for 4 hours. Scale bar corresponds to 2 µm. (C) Quantification of the peripheral to intracellular Can1-GFP fluorescence intensity ratio in cells presented in B. Error bars indicate s.d.m. (n = 15). (D) WT, ist2Δ, arg4Δ, arg4Δ ist2Δ, and arg4Δ ist2Δ cells expressing GFP-IST2 under control of the endogenous IST2 promoter from the his3Δ1 locus were spotted on YPD and HC media in five-fold serial dilutions starting from 2 OD600 and grown for 3 days at 25°C. (E) Relative growth of arg4Δ (black) and arg4Δ ist2Δ (white) cells in HC media with the indicated arginine concentrations. Note that the arginine concentration of standard HC medium is 95 µM. Pre-cultures grown in YPD medium for 18 hours were diluted to 0.05 OD600 into HC medium with the indicated arginine concentrations and grown for 16 hours. OD600 were normalized to WT grown in HC containing 95 µM arginine. Error bars indicate s.d.m. (n = 6). (F) CAN1 under control of pADH1 and pTEF1 promoters or empty plasmids were expressed in arg4Δ and arg4Δ ist2Δ cells from CEN plasmids. OD600 was normalized to arg4Δ transformed with empty plasmid. Error bars indicate s.d.m. (n = 12). Significance (unpaired, two-tailed student’s t-test) of p<0.005 and p<0.001 is indicated by double and triple asterisks. (TIF) [file pone.0085418.s004.tif]

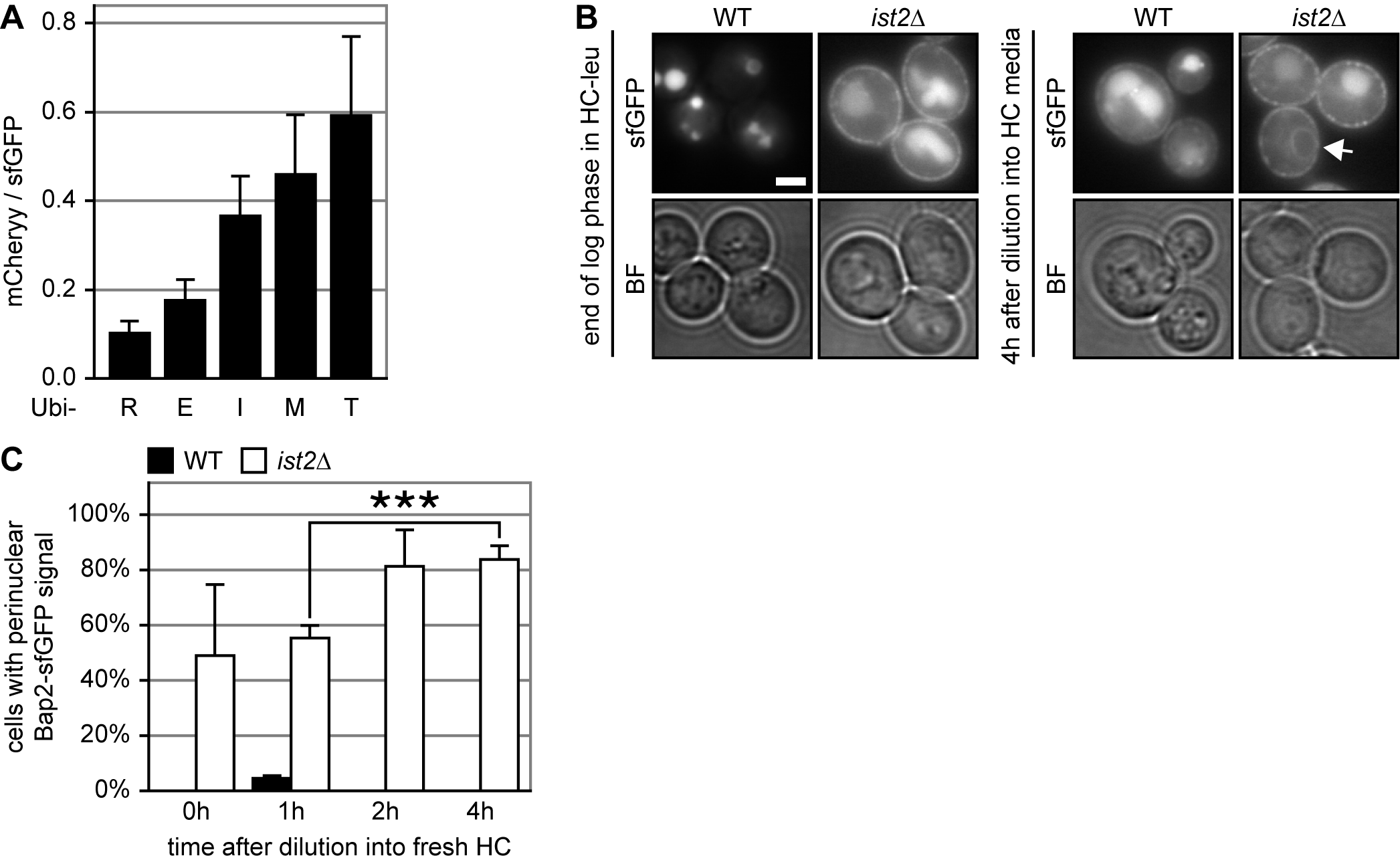

Supplement: Figure S5 — Bap2 accumulates in the perinuclear ER of ist2Δ. (A) mCherry/sfGFP fluorescence intensity ratios in cells expressing the indicated Ubi-X-mCherry-sfGFP constructs measured by epifluorescence microscopy. Mean mCherry and sfGFP intensities of a cytoplasmic region with a diameter of 537 pixels were quantified and are shown as mean+s.d.m (n = 100, except Ubi-I n = 69). Values for each construct are significantly different from neighbouring ones (p<0.001; unpaired, two-tailed student’s t-test). (B) Representative bright field (BF) and sfGFP images of Bap2-tFT expressed in WT and ist2Δ cells transformed with a LEU2 plasmid. Cells were grown for 18 hours in HC media without leucine followed by dilution into HC media containing leucine. Arrows in sfGFP images indicate perinuclear ER and scale bar corresponds to 2 µm. (C) Quantification of perinuclear ER Bap2-tFT signals (sfGFP channel) in WT and ist2Δ cells at different timepoints after dilution into fresh HC medium from 18 hours grown pre-cultures. Mean of four images with ≥12 cells chosen from bright field channel is shown. Error bars indicate s.d.m. and triple asterisks indicate significant difference (p<0.001; unpaired, two-tailed student’s t-test). (TIF) [file pone.0085418.s005.tif]
